# Genotyping and Cytology Co-Testing for Anal Cancer Screening: Exploratory Evaluation of Cumulative HPV Genotype Burden as a New Potential Marker for Risk Stratification

**DOI:** 10.3390/diagnostics16172729

**Published:** 2026-08-26

**Authors:** Cristina Sani, Claudia Giachini, Giampaolo Pompeo, Alessandra Mongia, Irene Paganini, Stephanie Pacella, Sara Galastri, Serena Giunti, Ornella Cutaia, Luigi Pisano, Giuseppe Gorini, Alessandro Senape, Martina Turco, Jacopo Farini, Filippo Caminati, Claudio Elbetti, Iacopo Giani, Nicola Pimpinelli, Stefania Cannistrà, Simonetta Bisanzi

**Affiliations:** 1 Regional Laboratory of Cancer Prevention, Institute for Cancer Research, Prevention and Oncological Network (ISPRO), Via Cosimo il Vecchio 2, 50139 Florence, Italy; c.sani@ispro.toscana.it (C.S.); c.giachini@ispro.toscana.it (C.G.); s.cannistra@ispro.toscana.it (S.C.); s.bisanzi@ispro.toscana.it (S.B.); 2 Section of Dermatology, Health Sciences Department, University of Florence, 50121 Florence, Italy; 3 Epidemiology Unit, Institute for Cancer Research, Prevention and Oncological Network (ISPRO), Via Cosimo il Vecchio 2, 50139 Florence, Italy; 4 Department of Public Health, Florence University, 50134 Florence, Italy; 5 Proctology Unit, USL Toscana Centro, 50121 Florence, Italy

**Keywords:** anal cancer screening, HPV, cumulative HPV genotype burden, anal cytology

## Abstract

**Background/Objectives**: Anal squamous cell carcinoma (SCCA) is a rare Human Papillomavirus (HPV)-related disease in the general population but its incidence is increasing among high-risk populations, i.e., men who have sex with men (MSM), HIV+ patients and women with a history of cervical/vulvar dysplasia. **The objectives of this study were**: (i) to investigate the prevalence of high-risk (HR) and low-risk (LR) anal HPV infections in a high-risk cohort; (ii) to correlate specific HPV genotypes with cytological and histological outcomes; and (iii) to assess the impact of cumulative HPV genotype burden. **Methods**: A retrospective study (2017–2022) was conducted on 550 high-risk patients. All patients underwent anal Pap tests (ThinPrep, Hologic) and HPV genotyping (Anyplex II HPV 28, Seegene), according to our screening protocol. High-Resolution Anoscopy (HRA) was performed on 430 patients; biopsy was performed in all cases with suspicious lesions. **Results**: The overall HR-HPV prevalence was 57.3%. Men showed a significantly higher prevalence of HR-HPV (59.9% vs. 47.4%) and cytological abnormalities (35.8% vs. 20.2%) compared to women. Notably, HPV 16, 58 and 45 accounted for 79% of AIN2+ (Anal Intraepithelial Neoplasia of grade 2 or higher) lesions. A significant cumulative HPV genotype burden effect, i.e., the number of HPV genotypes detected, was observed: patients with ≥3 HR-HPV types showed an increased risk of AIN2+ lesions (aOR 34.72; 95% CI 4.07–296.10) and AIN1+ lesions (aOR 16.26; 95% CI 4.54–58.30) respectively. The presence of ≥3 LR-HPV genotypes in HR-HPV-positive patients is associated with an increased risk of AIN1 lesions. **Conclusions**: HPV 16, 58 and 45 positivity and the cumulative HPV genotype burden could represent new potential indicators for risk stratification in high-risk populations. Future larger-scale prospective studies are needed to validate these preliminary results.

## 1. Introduction

Anal cancer is a relatively rare disease, but its incidence has been steadily increasing over the past few decades in some high-income countries, including those in Europe and the United States. The rise in cases is largely attributed to the increasing prevalence of Human Papillomavirus (HPV) infection, which is the primary risk factor for anal cancer especially among high-risk populations, i.e., people with an increased number of sexual partners and sexual habits, people living with HIV (PLWH), and men who have sex with men (MSM), regardless of their HIV status [1,2,3,4,5,6,7,8,9,10,11,12]. High-risk HPV infection (HR-HPV), which is associated with at least 90% of cancer cases, and immunosuppression are the main risk factors of this type of cancer.

The American Cancer Society estimates 9440 new cases of anal cancer in 2022 among the general population in the US, and the annual incidence continues to increase. The incidence among the general population (period 2001–2015) was 1.6/100,000 person-years; instead, among MSM living with HIV, it was estimated to be 89/100,000 person-years [13]. In Europe, the ICO/IARC 2023 report estimated 12,776 new cases of anal cancer in 2020 in the general population (1293 in Italy), with a slight increase compared to previous years. The incidence rate per 100,000 person-years was 1.05 and 0.66 for women and men respectively [14].

The most frequent histotype of anal cancer is squamous cell carcinoma (SCCA), representing about 80% of all diagnoses; others, such as adenocarcinoma and basal cell carcinoma, are relatively rare.

The mechanisms of pathogenesis and oncogenicity of HPV are the same as those underlying cervical cancer [15,16,17,18,19]. The ANCHOR study demonstrated that treatment of anal precancerous lesions significantly reduces the risk of progression to anal cancer in PLWH aged 35 and older [13]. This finding underscores the critical importance of early detection and treatment of anal precancerous lesions in preventing the development of invasive cancer.

HPV infection is a relevant factor in other anogenital cancers like anus, vulva, vagina and penis. In fact, HPV infection is commonly found in the anogenital tract of men and women with or without clinical lesions. Intraepithelial infection of the anal canal with different HPV genotypes can give rise to histologically distinct lesion types. More than 120 HPV types have been identified, classified as high-risk (HR-HPV) or low-risk (LR-HPV) based on their carcinogenic potential.

Anal HPV infections manifest as either single or multiple concurrent genotypes. The co-presence of multiple HPV genotypes may reflect cumulative immune evasion and sustained chronic inflammation induced by co-infecting genotypes, potentially favoring persistence, pre-neoplastic lesions onset and oncogenic progression. Nevertheless, to our knowledge, data specifically addressing the role of cumulative HPV genotype burden in anal precancerous lesions and anal cancer remain comparatively scarce, and largely derived from cohorts of HIV-infected men [20,21,22]. Evidence on this particular phenomenon in more heterogeneous high-risk populations, including HIV-negative MSM, women living with HIV or with prior HPV-related disease, is still limited.

It is well established that, similarly to cervical cancer onset, anal cancer is caused by HPV infection (in particular high-risk HPV types 16 and 18) and progresses through detectable precursor lesions called Anal Intraepithelial Neoplasia (AIN). These precancerous lesions can be identified via exfoliative sampling methods (liquid-based or conventional), tested in laboratory by an anal Pap test and/or HPV test and confirmed with High-Resolution Anoscopy (HRA) coupled with directed biopsies [23,24].

LR-HPV infection, especially HPV 6 and 11, contributes to 90% of the cases of anal warts/condylomatosis, which are traditionally considered benign lesions [25]. Several articles confirmed their primary role in warts while also acknowledging the rare, but possible, link to more serious lesions like precancerous lesions [26,27].

Although HPV vaccination has proven effective in reducing the incidence of anal intraepithelial neoplasia among HIV-positive patients, HPV-associated anal cancer remains a substantial burden, largely due to the high cost of vaccination.

Neoplasm of the anal canal is a rare disease in the general population, but it is more frequent in specific populations like MSM, PLWH patients and women with a history of high-grade squamous intraepithelial lesions (HSIL) or vulvar HPV cancer. Since in these target populations HPV prevalence is higher than in the general population, an appropriate screening protocol with a Pap test and/or HPV test is highly recommended in order to avoid an over-referral to HRA.

To date, several studies are ongoing but there is not a universally accepted protocol for the anal cancer screening and the anal Pap Test is not currently recommended for heterosexual men or women [28]. The International Anal Neoplasia Society (IANS) developed consensus guidelines to uniform the anal cancer screening algorithm among various high-risk populations: anal cytology or HR-HPV testing, used as a primary test (alone or with a triage test) and HR-HPV–cytology co-testing are two different strategies currently used for anal cancer screening that show acceptable performances [29].

All patients with symptoms should undergo a proctological examination including HRA. For asymptomatic patients belonging to the high-risk population, an initial HR-HPV test and an anal cytology allow to target patients at risk of AIN2+ lesions that are more likely to progress to cancer.

Data on the sensitivity and specificity of different tests or strategies for detecting anal cancer are highly variable and depend on several factors, including the study population (e.g., HIV-positive vs. general population). As reported by some colleagues [28], cytology performances for AIN2+ lesion detection is very effective (81% sensitivity and 62.4% specificity); the HPV test reported a higher sensitivity (91.9%) and a lower specificity (41.8%) and the co-testing strategy (cytology + HPV test) could be a valid option (93% sensitivity and 33.4% specificity) in order to detect more lesions in a hypothetical first round of screening.

Longitudinal studies that aim to evaluate the cumulative risks of anal precancer and cancer are needed to estimate how long negative tests guarantee protection. These studies are a requirement to provide evidence-based intervals for screening and management of laboratory test results.

We conducted a retrospective cohort study aimed to explore the prevalence of high-risk (HR) and low-risk (LR) anal HPV infections in a high-risk population; to correlate specific HPV genotypes with cytological and histological outcomes, focusing on sex-specific differences and to assess the impact of cumulative HPV genotype burden.

## 2. Materials and Methods

**Figure 1:**
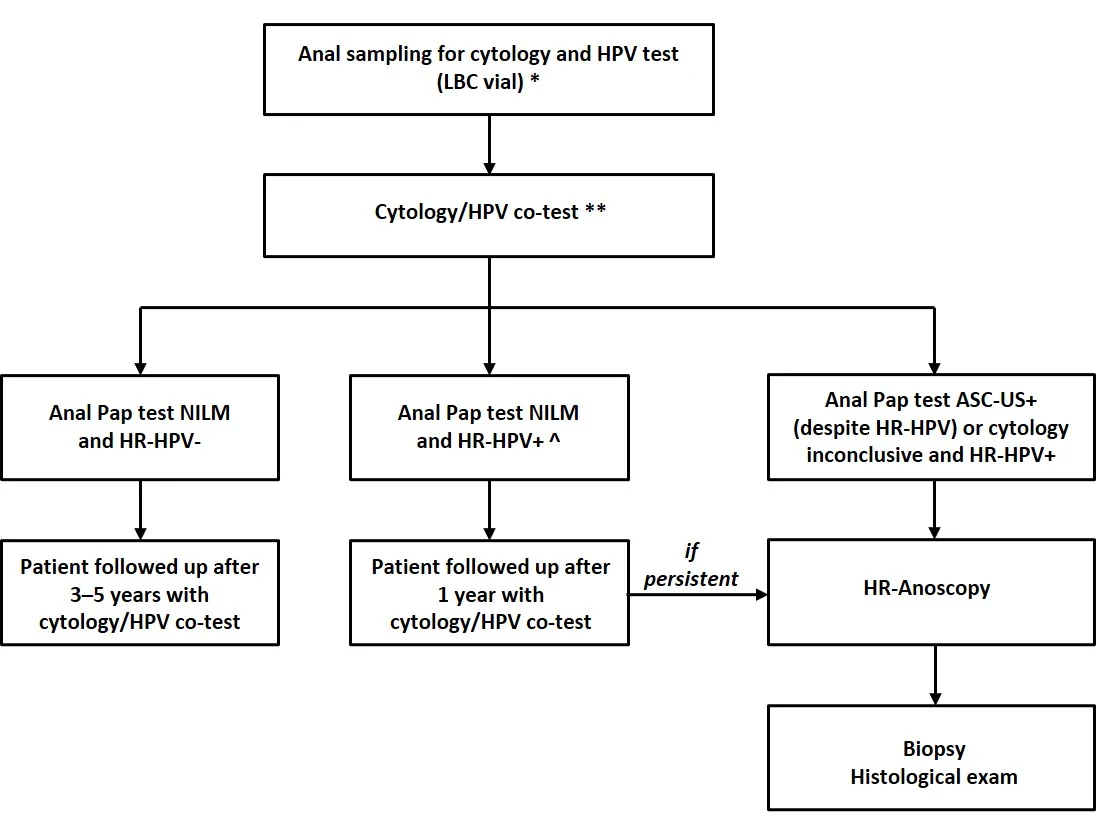
Anal cancer screening protocol actually performed in the district of Florence. LBC: Liquid-based cytology; NILM: negative for intraepithelial lesion or malignancy; ASC-US+: atypical squamous cells of undetermined significance (ASC-US) or more severe; * Sampling performed in STD center of Florence; ** cytology and HPV test are performed at ISPRO laboratory of Florence; ^ HR-HPV-positive patients who had never undergone an HR-anoscopy (HRA) and had not been previously followed at the Florence STD clinic were referred to HRA. If negative, they were scheduled for a one-year follow-up with a cytology–HPV co-test.

Since 2017 in the district of Florence (Italy), an internal screening protocol for anal cancer—based on cytology and HPV co-testing strategy (Figure 1)—has been performed in patients that are followed up at the Sexually Transmitted Diseases (STD) Center of the Dermatological Clinic of the University of Florence. We conducted a retrospective study, based on our screening database, considering the period 2017–2022. The inclusion criteria for enrolling patients were the same as in our previous article [30]: men who have sex with men (MSM), regardless of their HIV status, and women with at least one risk factor for anal cancer (HIV seropositivity, a history of anal condylomas, other HPV-related conditions, or any immunosuppression status); all patients were at least 18 years old and had already been screened at the STD center of Florence.

A total of 550 anal specimens (436 from men and 114 from women) were considered for our analysis, corresponding to 550 unique patients. A unique sampling for cytology and HPV genotyping, collected in Thinprep (Hologic, Marlborough, MA, USA), was performed. All samples were analyzed in the Regional Laboratory of Cancer Prevention at the Institute for Cancer Research, Prevention and Clinical Network (ISPRO) in Florence.

After the cytology test and HPV results (co-test), patients are followed up at the STD Center of Florence according on their internal screening protocol. When the HRA showed the presence of lesions characteristic of AIN, a biopsy was performed and sent to the Pathologic Anatomy Department of Careggi for the definitive histopathological diagnosis.

### 2.1. Anal Cytology

Liquid-based cytology (LBC) slides were prepared for all samples with an automated platform (Thin-Prep 5000 with Autoloader, Hologic, Marlborough, MA, USA). Slides were stained using an automated platform (Leica Multi-stainer, Leica Biosystems Nussloch GmbH, Nußloch, Germany) with Papanicolaou staining. Cytology was classified according to The Bethesda System for reporting cervical cytology (TBS 2001, subsequently updated in TBS 2014) [31], using the following categories: negative for intraepithelial lesion or malignancy (NILM), atypical squamous cells of undetermined significance (ASC-US), low-grade squamous intraepithelial lesion (LSIL), atypical squamous cells, cannot exclude high-grade squamous intraepithelial lesion (ASC-H), atypical glandular cells (AGC), high-grade squamous intraepithelial lesion (HSIL), and invasive carcinoma.

### 2.2. HPV Genotyping

The HPV genotyping test is based on detection of the viral genome by PCR on the same specimen used for anal cytology and preserved in ThinPrep. Before cytological slides were performed, an aliquot of specimen (1.5 mL) was used for molecular analysis with Anyplex II HPV 28 Detection test (Seegene, Seoul, Republic of Korea). This assay is a qualitative in vitro test for the detection of HPV in liquid-based cytology. The test is a multiplex real-time polymerase chain reaction assay that simultaneously allows the amplification, detection, and differentiation of 28 different HPV types, including 12 high-risk types (HPV 16, 18, 31, 33, 35, 39, 45, 51, 52, 56, 58, 59) and 16 classified as intermediate or low-risk oncogenic types (HPV 6, 11, 40, 42, 43, 44, 54, 61, 26, 53, 66, 69, 70, 73, 82), as well as internal control. It is an extremely sensitive test with a detection limit of 50 copies/reaction.

### 2.3. High-Resolution Anoscopy and Treatment Protocol

HRA was typically performed without prior bowel preparation, with the patient positioned in lithotomy [30,32]. A disposable anoscope was inserted into the anal canal using lidocaine gel for lubrication, followed by insertion of a gauze soaked in 5% acetic acid solution (Bio Optica, Milan, Italy), which was left in place for up to 1 min. After the application of the acetic acid solution, the anoscopy was performed with the aid of an optical colposcope taking care to evaluate, above all, the anal transformation zone (AnTZ), smoothing the mucosa and visualizing the crypts. Other target areas for observation are the squamocolumnar junction (SCJ), the anal canal (AC, including the anal verge), and the perianal region using external HRA. If lesions characteristic of dysplastic lesions called “aceto-white changes” were seen, thanks to image magnification, the morphological features are described: contour, surface pattern, vascular pattern. At the conclusion of the examination, a 3% Lugol iodine solution (Sigma-Aldrich, Burlington, MA, USA) using a cotton swab was applied selectively on the lesion. Lesions considered highly suspicious of being an AIN2+ typically turn to a yellowish color (Lugol-negative) and show specific vascular alterations such as punctations, mosaic or atypical vascular pattern. A biopsy was then performed and sent to the Pathologic Anatomy Unit for histopathological diagnosis.

According to the lower anogenital squamous terminology guidelines, biopsies were classified as follows: NEG (negative), AIN1 (anal intraepithelial neoplasia grade 1, LSIL) or AIN2+ (AIN2, AIN3, or carcinoma in situ, HSIL).

Patients diagnosed with AIN2 or higher-grade lesions (AIN2+) are referred for treatment, consistent with current clinical practice. Our follow-up protocol also haves practical importance for the patients with AIN1 histological diagnosis. Although this type of histological lesion is not routinely treated, these patients are monitored more closely over time compared to patients with negative HRA or histology results, according to the follow-up protocol. If repeated, for our analysis the worst HRA and histological results within 12 months from the first co-testing sampling were considered.

### 2.4. Statistical

Associations between categorical outcomes were assessed via Fisher’s exact test, reporting Odds Ratios (ORs) and adjusted Odds Ratio (aOR) and 95% confidence intervals (CIs). Statistical significance was defined as a *p*-value < 0.05. Multivariable logistic regression models were constructed to assess the association between cumulative HPV genotype burden and AIN1+/AIN2+ lesions using AIN1+ vs. AIN1- or AIN2+ vs. AIN2- as dependent variables, adjusting for age, sex, HIV status, and HPV vaccination.

All statistical analyses were performed using Stata software (version. 15.0, StataCorp, College Station, TX, USA).

**Figure 2:**
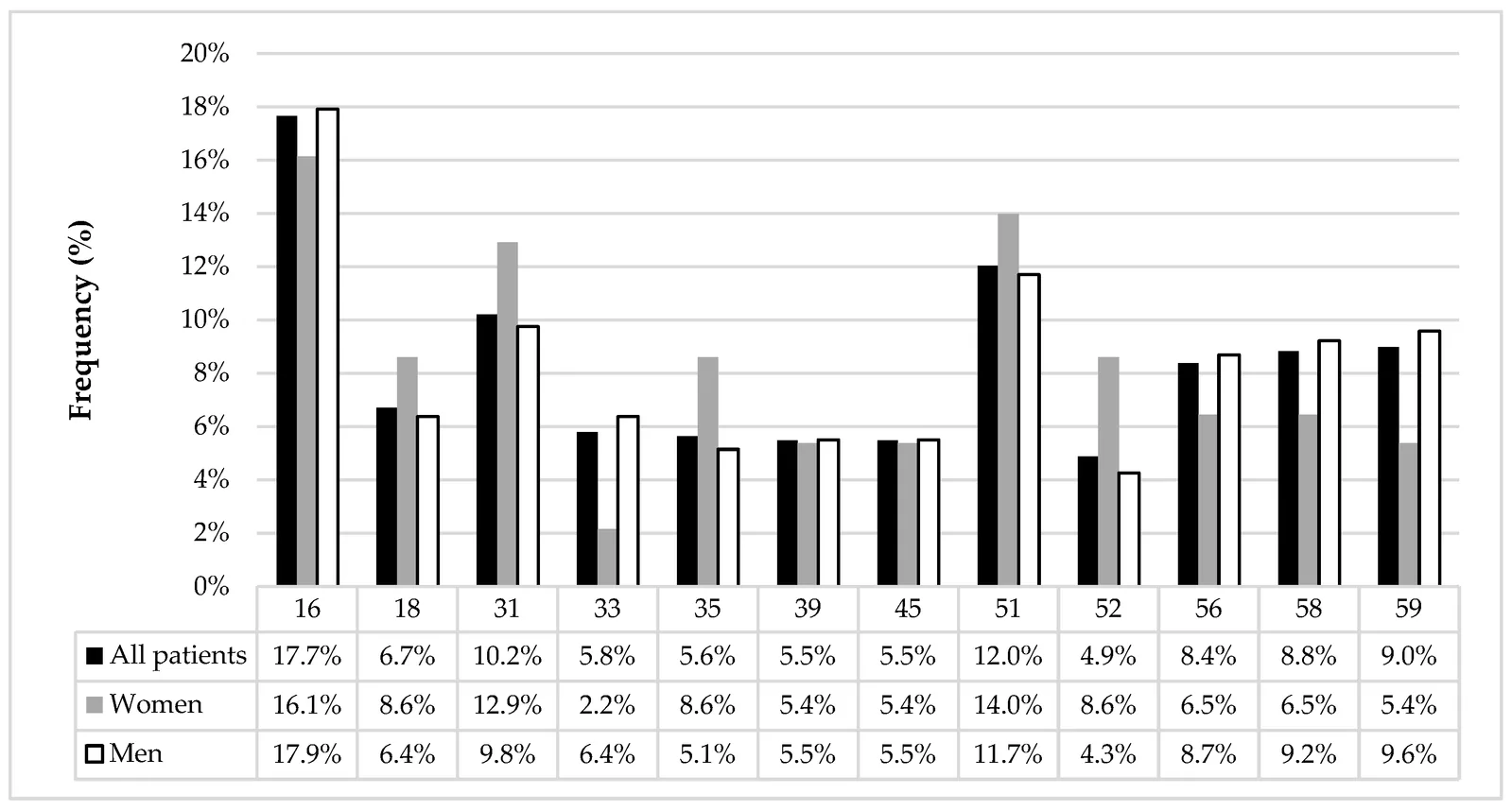
Distribution of HR-HPV genotypes among all detected infections (*n* = 657: 93 in women, 564 in men). For each genotype, percentages represent the proportion of HPV type-specific detections relative to the total number of infections in the corresponding group (all patients, women and men); as multiple genotypes could be detected in the same patient, denominators reflect infections rather than individual patients.

**Figure 3:**
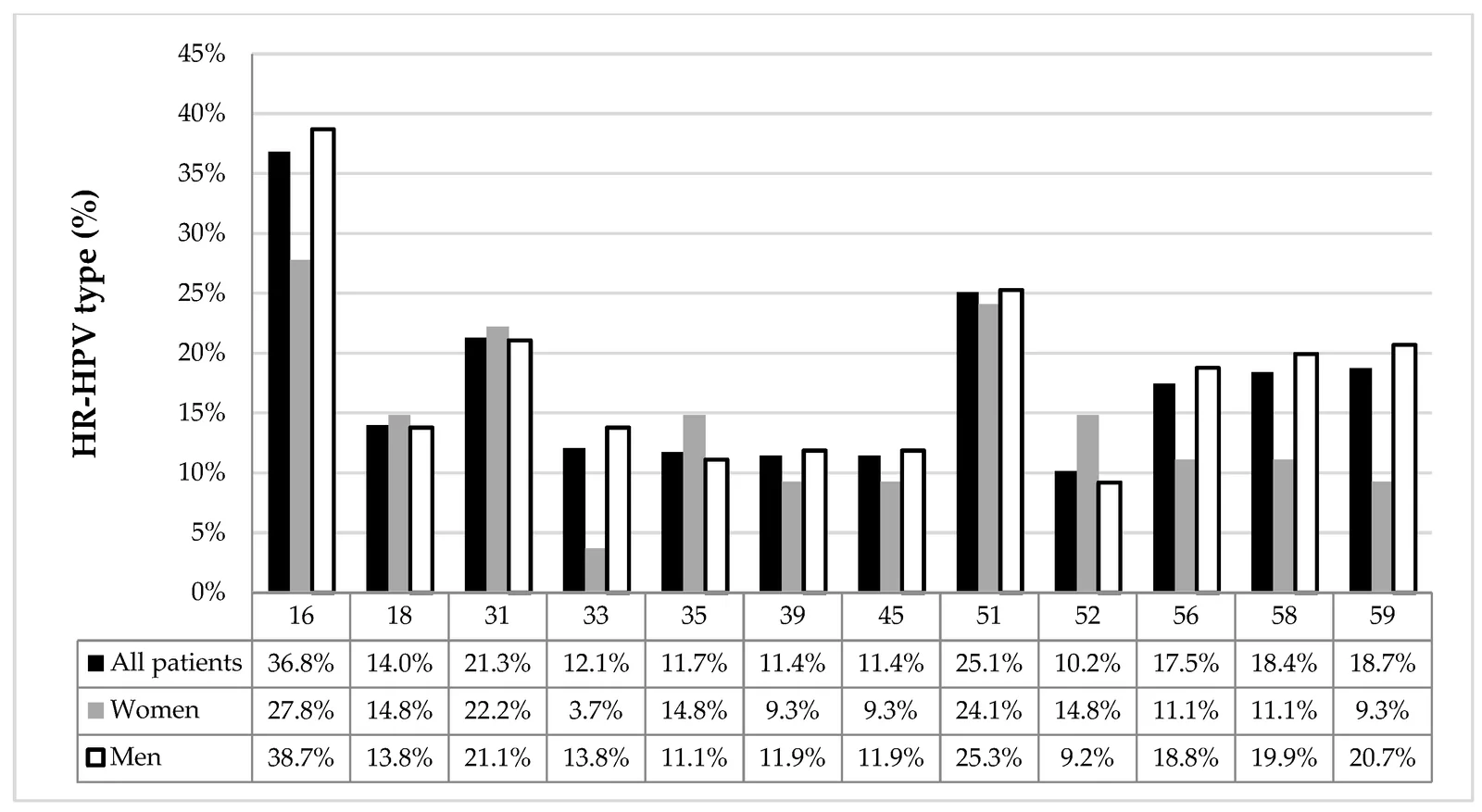
Prevalence of each HR-HPV genotype among HR-HPV-positive patients (*n* = 315: 54 women, 261 men). For each genotype, the percentage represents the proportion of patients positive for that specific HPV type relative to the total number of HR-HPV-positive patients in the corresponding group (all patients, women, or men). Several patients tested positive for multiple HPV genotypes concurrently, so percentages across genotypes are not mutually exclusive and do not sum to 100%.

**Figure 4:**
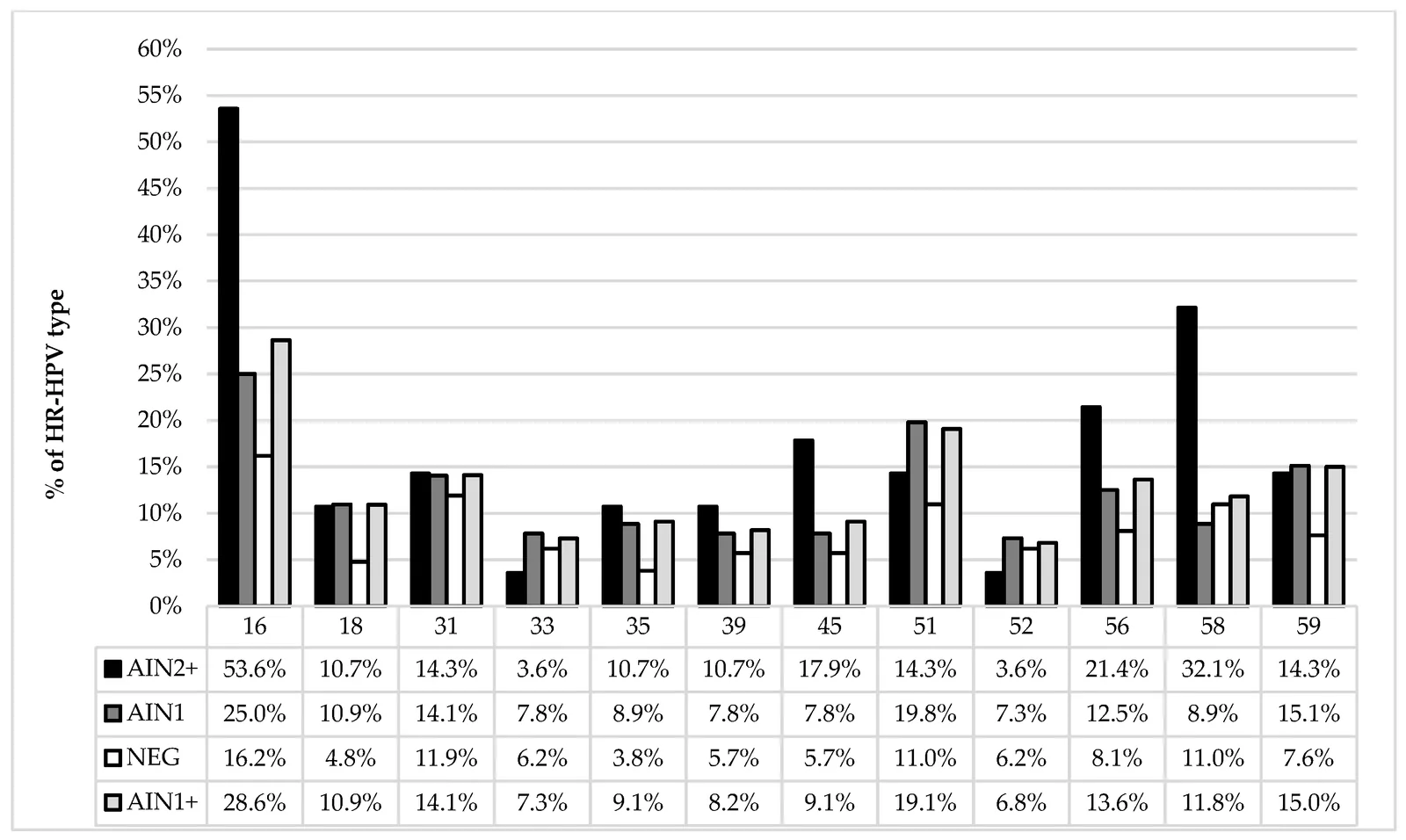
Frequency of individual HR-HPV genotypes across HRA-histological outcome categories (total *n* = 430): negative (NEG, *n* = 210), AIN1 (*n* = 192), AIN2+ (*n* = 28), and AIN1+ [AIN grade 1 or higher, i.e., AIN1 and AIN2+] (*n* = 220). For each histological category, the percentage represents the proportion of patients within that group tested positive for the specific HR-HPV genotype, using the total number of patients in the corresponding category as the denominator. As patients could be positive for multiple genotypes concurrently, percentages within each category are not mutually exclusive and do not sum to 100%.

**Figure 5:**
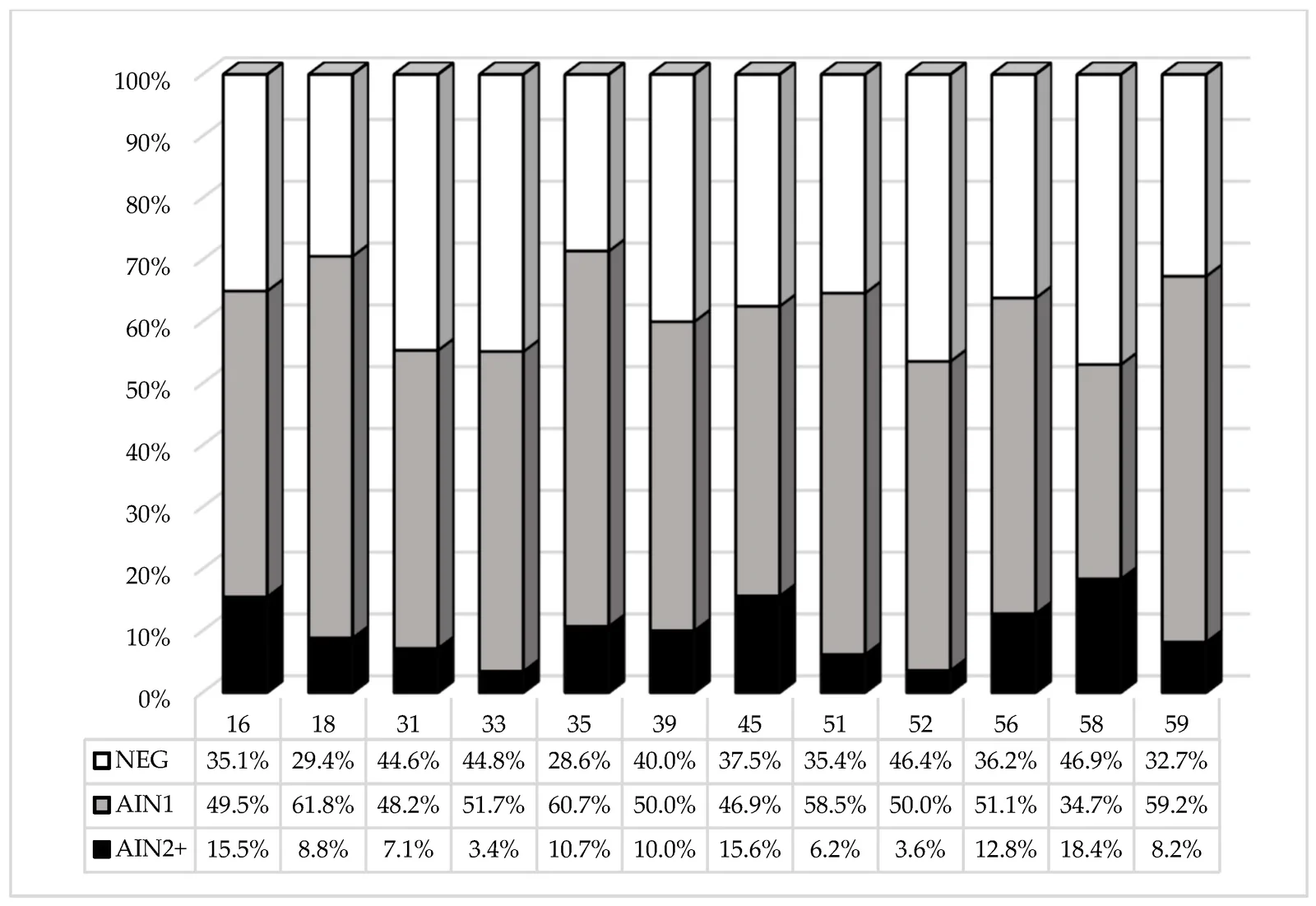
Distribution of HRA-histological outcomes (Negative, AIN1, AIN2+) across HR-HPV genotypes, presented as a 100% stacked bar chart, to characterize the spectrum of histological lesions associated with each HPV genotype and highlight potential differences in AIN2+ risk across HR-HPV types. A total of 544 HR-HPV infections were detected among the 430 patients with an available HRA-histological outcome (multiple HR-HPV infections were possible per patient). For each HR-HPV genotype, the bar represents the proportion of HPV genotype-specific infections associated with each HRA-histological outcome, using the total number of infections of that genotype as the denominator.

Percentages in the HPV distribution figures (Figure 2, Figure 3, Figure 4 and Figure 5) were calculated as follows, using different denominators. Figure 2 shows genotype frequencies relative to the total number of HR-HPV infections (*n* = 657), as patients could have multiple concurrent genotypes. Figure 3 and Figure 4 show genotype-specific prevalence relative to the total number of HR-HPV-positive patients (*n* = 315) and to the number of patients in each HRA-histological category (Negative, AIN1, AIN2+, AIN1+; total *n* = 430), respectively; in both cases, values are not mutually exclusive due to possible multi-genotype infections. Figure 5 shows the spectrum of HRA-histological lesions associated with each HR-HPV genotype, presented as a 100% stacked bar chart, to characterize which genotypes might be associated with a greater risk of AIN2+. Among the 430 patients with an available HRA-histological outcome (Negative, AIN1, AIN2+), 544 HR-HPV infections were detected (multiple concurrent genotype infections were possible within the same patient). For each genotype, the HRA-histological outcome distribution was calculated using the total number of infections of that specific genotype as the denominator.

## 3. Results

A total of 550 individuals were included in the study (see Table 1 for detailed characteristics).

The results of co-testing (Pap Test and HPV genotyping) for the 550 anal samples are detailed in Table 2. The rate of HPV positivity in the whole study population was 57.3% and was significantly higher (*p* = 0.02) in men (59.9%) than in women (47.4%). The rate of cytological abnormalities was 32.5% and was significantly higher (*p* = 0.001) in men (35.8%) than in women (20.2%).

The correlation between HPV status and cytological categories is reported in Table 2. Among the 315 HR-HPV+ patients (with one or more HR-HPV types, resulting in a total of 657 HR-HPV infections), 136 (43.2%) had cytological abnormalities (ASC-US or more severe; ASC-US+). ASC-H and HSIL categories were found only in HR-HPV+ patients. NILM results were more frequent in HR-HPV+ women than in men. A more detailed report about the correlation between HPV status/cytological result is reported in Table 3.

The distribution of HR-HPV genotypes was assessed on the total number of detected infections (*n* = 657), including co-infections, and was similar in women and in men (Figure 2). The most frequent HR-HPV types were HPV 16, HPV 51 and HPV 31, representing 17.7%, 12.0% and 10.2% of infections, respectively.

The prevalence of different HR-HPV types of infection was calculated on the total number of HR-HPV+ patients and in women and men separately (Figure 3). The results were similar between men and women (except for HPV 33; *p* = 0.038).

Regarding the number of HPV co-infections, some patients had co-infections up to seven and nine types for HR-HPV and LR-HPV respectively (Table 4); this aspect particularly affects men compared to women; the incidence of co-infection with three or more HR-HPV was significantly higher (*p* = 0.01) in men (88/261, 33.7%) than in women (9/54, 16.7%). Comparable results were found considering the number of LR-HPV co-infections in HR-HPV-positive patients, which was significantly higher (*p* = 0.04) in men (106/261, 40.6%) than in women (14/54, 25.9%).

According to the anal cancer screening protocol (Figure 1), HRA was performed in 430 patients (93 women and 337 men). A biopsy was performed in all cases where HRA highlighted suspicious lesions. The relationship between HPV status and HRA-histological outcomes is presented in Table 5.

Among individuals with a HR-HPV infection, the frequency of AIN2+ was 10.2% (27/265) and the results were similar when comparing women and men. In LR-HPV-positive patients, AIN1 and negative HRA-histology showed a similar frequency and no cases of AIN2+ were observed.

As expected, all cases with AIN2+ were HR-HPV-positive patients with the exception of one case, which nevertheless exhibited positive cytology (AGC, atypical glandular cells).

Although AIN1 lesions were mostly associated with HR-HPV infection (66.7%), a considerable proportion of cases presented only LR-HPV infection (32.3%). Considering all lesions as a unique category (AIN1+), HR-HPV+ men showed a higher prevalence of AIN1+ compared with HR-HPV+ women (60.5% versus 48.9%) with a statistically significant difference (*p* < 0.01).

To investigate the role of HR-HPV-specific genotypes, we evaluated the frequency of HR-HPV types across different HRA-histological outcomes (Figure 4), in order to identify which HPV genotypes are preferentially associated with AIN lesions. HPV16 was the most frequent in AIN2+ lesions (15/28; 53.6%), followed by HPV58 (9/28; 32.1%) and HPV 56 (6/28; 21.4%).

In order to characterize the spectrum of HRA-histological lesions associated with each HPV genotype and provide more insight into the biological effect of different HR-HPV types (i.e., which HR-HPV types could be correlated with a greater risk of developing AIN2+ lesions), we assessed the distribution of HRA-histological outcomes across HR-HPV genotypes (Figure 5).

AIN2+ resulted more frequently in patients with HPV16, HPV58 and HPV45 infection: 15/97 (15.5%), 9/49 (18.4%) and 5/32 (15.6%) respectively.

Furthermore, we performed a multivariate analysis using a logistic regression model to verify the association between these three HR-HPV genotypes (16, 45, and 58) and AIN1+ and AIN2+ lesions, using HR-HPV-positive patients who were negative for these specific genotypes as the reference group (Table 6).

Our analysis confirms that HPV 16 is the genotype most strongly associated with high-grade disease (AIN2+) with an aOR 4.85 (95% CI 2.14–11.02), and this is consistent with HPV 16’s established oncogenic effect already demonstrated in the literature. HPV 45 shows a divergent pattern: no significant association with AIN1+ (aOR 1.57, 95% CI 0.74–3.36), but a significant association with AIN2+ lesions (aOR 3.11, 95% CI 1.05–9.19). HPV58 shows the same qualitative pattern as HPV 45: no association with AIN1+ but a significant association with AIN2+ lesions (aOR 4.33, 95% CI 1.70–11.01). Compared with subjects negative for all three genotypes, those positive for at least one of HPV 16, 45, or 58 had a significantly increased risk of both AIN1+ (aOR 1.76, 95% CI 1.15–2.70) and, strongly, AIN2+ (aOR 8.58, 95% CI 3.24–22.70).

When stratifying by the total number of concurrent HR-HPV genotypes among subjects positive for at least one of HPV 16, 45 or 58 (Table 7), a cumulative HPV genotype burden effect was observed for AIN1+ with ≥3 genotypes (aOR 3.00, 95% CI 1.61–5.58). For AIN2+, the risk increase was already substantial with 1–2 additional genotypes (aOR 8.53, 95% CI 3.04–24.00).

Given the high prevalence of HR-HPV and HR/LR-HPV co-infections within the screened cohort, we sought to analyze the potential role of these co-infections in association with AIN1+ and AIN2+ lesions, in order to evaluate a possible cumulative effect of HPV genotype burden.

Table 8 reports the Odds Ratios (ORs) and corresponding 95% confidence intervals for the cumulative LR and HR-HPV genotype burden effect on AIN1+ lesions. We found that risk increased progressively with higher numbers of HR-HPV genotypes within each LR-HPV stratum, and similarly increased with higher numbers of LR-HPV genotypes within each HR-HPV stratum. Subjects with ≥3 LR-HPV genotypes (in the absence of HR-HPV) showed an OR of 9.82 (95% CI: 2.49–38.69) compared with the reference group, while those with ≥3 LR-HPV genotypes combined with ≥3 HR-HPV genotypes showed the highest risk, with an OR of 41.15 (95% CI: 11.84–142.93). Notably, even a low burden of LR-HPV genotypes (1–2) combined with high HR-HPV multiplicity (≥3) yielded a substantial OR of 16.26 (95% CI: 4.54–58.30), suggesting a possible synergistic or additive effect between LR and HR-HPV multiple infections.

Table 9 shows the association between cumulative HR-HPV and LR-HPV genotype burden and AIN2+ lesions. Among all subjects, increasing numbers of HR-HPV genotypes were strongly associated with AIN2+, compared with subjects negative for HR-HPV, indicating a relationship between cumulative HR HPV genotype burden and the risk of AIN2+ lesions.

In contrast, when the analysis was restricted to HR-HPV-positive subjects and stratified by the additional presence of LR-HPV genotypes, no significant association with AIN2+ emerged. These findings suggest that AIN2+ risk is primarily associated with cumulative HR-HPV genotype burden, while concomitant LR-HPV infection does not appear to independently modify this risk in HR HPV+ patients.

## 4. Discussion

According to the International Anal Neoplasia Society (IANS) consensus guidelines, anal cytology or HR-HPV testing—either alone, as a triage, or in co-testing—is an acceptable strategy for anal cancer screening.

Since 2017, a screening protocol for SCCA based on an anal cytology and HPV co-testing strategy (Figure 1) has been applied to high-risk patients attending the Sexually Transmitted Diseases (STD) Center of the Dermatological Clinic of the Piero Palagi Hospital. Our previous work demonstrated that co-testing achieved 100% sensitivity and 100% NPV for AIN2+ lesions, establishing it as a highly reliable approach [30].

This retrospective study (2017–2022) was designed to investigate the prevalence of high-risk (HR) and low-risk (LR) anal HPV infections within a high-risk population. Additionally, we aimed to correlate distinct HPV genotypes with cytological and histological outcomes and also assess the possible impact of cumulative HPV genotype burden and its association with AIN lesions.

The prevalence of HR-HPV infection in our study population (*n* = 550) was 57.3%, confirming a high frequency of this sexually transmitted infection within this high-risk cohort. Our findings reveal a significant sex difference, with a higher prevalence of both HR-HPV infection (59.9% vs. 47.4%) and cytological abnormalities (35.8% vs. 20.2%) in men compared to women. This gap is further emphasized by the significantly higher incidence of multiple HR-HPV co-infections (three or more genotypes) in the male subgroup (33.7% vs. 16.7%; *p* = 0.01). Notably, a similar trend was observed for LR-HPV co-infections among HR-HPV-positive patients, which were significantly more frequent in men than in women (40.6% vs. 25.9%; *p* = 0.04). These results could reflect differences in sexual behavior rather than differences in terms of biological susceptibility of the anal mucosa, or divergent local immune responses between sexes.

Despite these differences in HPV prevalence, the distribution of specific genotypes was largely consistent across sexes. HPV 16, 51, and 31 were the most frequent HR-HPV types identified in both groups (Figure 2), aligning with previous literature.

Regarding clinical outcomes, High-Resolution Anoscopy (HRA) was performed in 430 patients (93 women and 337 men), according to our anal cancer screening protocol (Figure 1). Our findings confirm a strong correlation between HPV status and histological severity: among individuals with HR-HPV infection, the frequency of high-grade lesions (AIN2+) was 10.2%, with no significant difference observed between women and men; conversely, in patients with only LR-HPV infections, no cases of AIN2+ were detected, and AIN1 showed a similar frequency to negative histology. Notably, nearly all AIN2+ cases (96.4%) were HR-HPV-positive, with the exception of one case, which tested HPV-negative and presented a cytological result of atypical glandular cells (AGC), yielding an overall Positive Predictive Value (PPV) for our screening strategy of 10.6%. A key aspect of our analysis is the potential role of the cumulative HPV genotype burden in association with AIN lesions, considering “HPV burden” refers to the number of HPV genotypes detected, not viral load. Our data suggest that the risk of presenting AIN1+ lesions increases significantly with the number of HPV co-infecting types (Table 8), suggesting a potential synergistic effect between LR-HPV and HR-HPV infections. The presence of three or more LR-HPV types (≥3) in HR-HPV-positive patients significantly increases the risk of AIN1+ lesions (aOR 41.15; 95% CI: 11.84–142.93) compared to patients infected only with multiple HR-HPV infections (aOR: 16.26; 95% CI 4.54–58.30). Moving on the possible association of cumulative HPV genotype burden with AIN2+ lesions (Table 9), patients positive to three or more HR-HPV types (≥3) showed a significantly higher prevalence of AIN2+ (aOR: 34.72; 95% CI 4.07–296.10). Regarding the additional presence of LR-HPV genotypes in HR-HPV-positive patients (cumulative LR-HPV and HR-HPV genotype burden), no significant association or cumulative effect with AIN2+ lesions emerged.

Genotype-specific analysis further reveals that HPV 16, 45 and 58 are significantly associated with high-grade lesions in this cohort, accounting for 79% of all AIN2+ lesions. Our data confirm HPV 16 as the genotype most consistently and strongly associated with anal precancerous lesions, driving risk at both the low-grade (AIN1) and high-grade (AIN2+) lesions (Table 6), consistent with its recognized oncogenic predominance. In contrast, HPV45 and HPV58 appear to be not associated with an increased likelihood of low-grade lesion (AIN1), but both significantly increased the risk of high-grade lesions (AIN2+). The pooled analysis further shows that simply being positive for at least one of these three genotypes confers a markedly higher risk of AIN2+ (aOR 8.58, 95% CI 3.24–22.70); this restricted genotype panel could be useful for identifying patients at risk of high-grade, clinically relevant disease rather than any HPV-related abnormality.

The analysis of cumulative HR-HPV genotype burden in patients positive to HPV 16, 45 or 58 (Table 7) indicates a possible effect on AIN1+, where risk increases progressively and reaches significance only with ≥3 concurrent HR-HPV genotypes (aOR 3.00, 95% CI 1.61–5.58). For AIN2+, in contrast, risk was already markedly elevated with just one additional HR-HPV genotype (aOR 8.53, 95% CI 3.04–24.00) and did not rise further with ≥3 HR-HPV genotypes.

It is important to underline that a high number of concurrent HR-HPV and/or LR-HPV genotypes may reflect greater sexual exposure, higher-risk sexual behavior, or underlying immune dysfunction, rather than a direct biological effect of multiple concurrent infections.

Genotype-specific analysis further reveals that, while HPV 16 is globally recognized as the most oncogenic HPV type, the high frequency registered in our cohort for HPV 58 and the prevalence of AIN2+ patients infected with this genotype are surely more unusual in other European countries, North America, and Oceania, but it has been more frequently reported in Asian and South American countries [14,15,16,17,18,19,,20,21]. These data may suggest that HPV 16, 45 and 58 positivity could represent potential markers for risk stratification in our population, hypothesizing a more intensive follow-up protocol, even after NILM cytological results.

Both the usefulness of cumulative HPV genotype burden and the stratification of the population with a higher risk of AIN2+ lesions onset require validation in larger prospective cohorts.

Our study is characterized by several strengths. The sample size is relatively large (550 patients) and patients referred for second-level assessment underwent HRA, which allowed histological sampling to be obtained consistently across the cohort. The use of targeted biopsies further contributed to a high degree of diagnostic accuracy. Moreover, we introduce the concept of “cumulative HPV genotype burden” (i.e., the presence of multiple concurrent HPV infections) as a potentially novel variable for screening protocols, particularly in view of its possible role as a risk stratification tool. Given the elevated prevalence of HPV positivity and the distinctive features of this high-risk population, a risk stratification approach based on specific HR-HPV genotypes may prove particularly valuable.

Some limitations should also be acknowledged. The number of AIN2+ lesions was relatively small (*n* = 28), which may limit the statistical power of subgroup analyses involving this outcome. Detailed clinical data on HIV-positive patients, including specific CD4 cell counts and the exact nature of immunosuppression, were not available, preventing their inclusion in the multivariable analysis. Furthermore, histological diagnosis was not obtained uniformly across the entire cohort. Finally, in some statistical analyses, AIN1+ was used as a surrogate outcome; although AIN1 lesions are known to have a higher likelihood of spontaneous regression compared with high-grade AIN2+ lesions, and therefore do not carry the same clinical significance, this outcome still retains practical relevance for our screening protocol.

## 5. Conclusions

The goal of an effective screening strategy should extend beyond the early detection of cancer, aiming instead at its prevention through the identification and treatment of high-grade precancerous lesions (AIN2 and AIN3), together with close follow-up of patients diagnosed with AIN1. Our findings indicate that the combination of cytology and HPV co-testing can effectively identify patients at increased risk of AIN lesions.

Given the high prevalence of HR-HPV and the high rate of cytological positivity, it is essential to risk-stratify the screened population to avoid over-referral to HRA. A crucial finding of this study is that the cumulative HR-HPV genotype burden might be associated with an increased rate of cytological abnormalities and/or to AIN2+ lesions and that LR and HR-HPV genotypes could act in a synergistic way in AIN1 lesions onset. These results underscore the importance of performing extended genotyping (including LR-HPV types) in anal cancer screening. Our data also demonstrate that a periodic monitoring of the most frequent and prevalent HPV types in patients with AIN2+ lesions is a viable strategy (i.e., HPV 16, 58 and 45).

We could hypothesize subtle adjustments to our follow-up strategy for HR-HPV-positive patients, including: a closer follow-up for patients with AIN1 lesions tested positive to multiple HR-HPV infections or to at least one of the “higher risk” genotypes, HPV 16, 45 or 58; a more intensive follow-up for patients with low-grade cytology (ASC-US or LSIL) who tested positive for HPV 16, 45 or 58; a less intensive follow-up for patients with negative cytology but positive for only one HR-HPV type excluding 16, 45 and 58.

Additionally, the introduction of prognostic markers such as p16/Ki-67 dual-staining is currently under investigation in our cohort; it could help optimize follow-up protocols due to its better performance both for clinical sensitivity and specificity for AIN2+ lesions, allowing for longer testing intervals or referral of patients for immediate anoscopy regardless of the cytologic result.

Further assessments involving a larger patient cohort and an extended follow-up period are warranted to confirm these preliminary findings..

## Figures and Tables

**Table 1:** Demographic and clinical characteristics of the study population (*n* = 550).

Characteristic	*n* (%)
Total Population	550 (100.0)
Sex	
Male	436 (79.3)
Female	114 (20.7)
Age groups	
18–29	95
30–39	131
40–49	130
50–59	136
60+	58
Sexual orientation	
Homosexual	354 (64.4)
Heterosexual	169 (30.7)
Bisexual	18 (3.3)
Unknown	9 (1.6)
HIV status	
Positive	140 (25.5)
Negative	400 (72.7)
Unknown	10 (1.8)
HPV vaccination status	
Vaccinated	66 (12.0)
Unvaccinated	484 (88.0)

**Table 2:** HPV status (detailed as HR-HPV+, LR-HPV+ and HPV negative) and cytology results in the study population (categorized into women, men and all patients). * It may include co-infection with LR HPV; ** LR-HPV infection only.

		Women		Men		All Patients	
		n	%	n	%	n	%
HPV status	HR-HPV+ *	54	47.3%	261	59.9%	315	57.3%
	LR-HPV+ **	41	36.0%	114	26.1%	155	28.2%
	HPV negative	19	16.7%	61	14.0%	80	14.5%
	Total	114	100.0%	436	100.0%	550	100.0%
Cytology result	NILM	81	71.0%	253	58.0%	334	60.7%
	ASC-US	10	8.8%	60	13.8%	70	12.7%
	LSIL	11	9.6%	81	18.6%	92	16.7%
	ASC-H	1	0.9%	5	1.1%	6	1.1%
	HSIL	1	0.9%	7	1.6%	8	1.5%
	AGC		0.0%	3	0.7%	3	0.6%
	Inconclusive	10	8.8%	27	6.2%	37	6.7%
	Total	114	100.0%	436	100.0%	550	100.0%

**Table 3:** Correlation between HPV status and cytology results in the study population and in women versus men. * It may include co-infection with LR-HPV; ** LR-HPV infection only.

	HPV Status						
	Cytology Result	HR-HPV *		LR-HPV **		HPV Negative	
		n	%	n	%	n	%
All patients	NILM	165	52.4%	102	65.8%	67	83.8%
	ASC-US+	136	43.1%	40	25.8%	3	3.8%
	ASC-US	50	15.9%	18	11.6%	2	2.5%
	LSIL	70	22.2%	22	14.2%	0	0.0%
	ASC-H	6	1.9%	0	0.0%	0	0.0%
	HSIL	8	2.5%	0	0.0%	0	0.0%
	AGC	2	0.6%	0	0.0%	1	1.3%
	Inconclusive	14	4.4%	13	8.4%	10	12.5%
	Total	315	100.0%	155	100.0%	80	100.0%
Women	NILM	37	68.5%	31	75.6%	13	68.4%
	ASC-US+	15	27.8%	7	17.1%	1	5.3%
	ASC-US	6	11.1%	3	7.3%	1	5.3%
	LSIL	7	13.0%	4	9.8%	0	0.0%
	ASC-H	1	1.9%	0	0.0%	0	0.0%
	HSIL	1	1.9%	0	0.0%	0	0.0%
	Inconclusive	2	3.7%	3	7.3%	5	26.3%
	Total	54	100.0%	41	100.0%	19	100.0%
Men	NILM	128	49.0%	71	62.3%	54	88.5%
	ASC-US+	121	46.3%	33	29.0%	2	3.2%
	ASC-US	44	16.9%	15	13.2%	1	1.6%
	LSIL	63	24.1%	18	15.8%	0	0.0%
	ASC-H	5	1.9%	0	0.0%	0	0.0%
	HSIL	7	2.7%	0	0.0%	0	0.0%
	AGC	2	0.8%	0	0.0%	1	1.6%
	Inconclusive	12	4.6%	10	8.8%	5	8.2%
	Total	261	100.0%	114	100.0%	61	100.0%

**Table 4:** HR-HPV and LR-HPV infection number in HR-HPV-positive patients.

**HR-HPV Number**	**Men**		**Women**		**All Patients**	
	**n**	**%**	**n**	**%**	**n**	**%**
1	106	42.2%	27	50.0%	133	42.2%
2	67	27.0%	18	33.3%	85	27.0%
3	48	17.5%	7	13.0%	55	17.5%
4	26	8.6%	1	1.9%	27	8.6%
5	9	3.2%	1	1.9%	10	3.2%
6	4	1.3%	-	0.0%	4	1.3%
7	1	0.3%	-	0.0%	1	0.3%
Total	261	100.0%	54	100.0%	315	100.0%
**LR-HPV Number in HR-HPV+ Patients**	**Men**		**Women**		**All Patients**	
	**n**	**%**	**n**	**%**	**n**	**%**
0	34	13.0%	13	24.1%	47	14.9%
1	58	22.2%	12	22.2%	70	22.2%
2	63	24.1%	15	27.8%	78	24.8%
3	40	15.3%	7	13.0%	47	14.9%
4	29	11.1%	6	11.1%	35	11.1%
5	21	8.0%	-	-	21	6.7%
6	7	2.7%	1	1.9%	8	2.5%
7	6	2.3%	-	-	6	1.9%
8	1	0.4%	-	-	1	0.3%
9	2	0.8%	-	-	2	0.6%
Total	261	100.0 %	54	100.0%	315	100.0%

**Table 5:** Correlation between HPV status (HR, LR and negative) and HRA-histological results (AIN1, AIN2+, negative) among men, women and all patients. * May include co-infection with LR HPV; ** LR HPV infection only. ^#^ Biopsy was performed when HRA showed a suspicion of AIN lesion.

		Negative ^#^		AIN1		AIN2+		TOTAL	
	HPV Status	n	%	n	%	n	%	n	%
All patients	HR-HPV+ *	110	52.4%	128	66.7%	27	96.4%	265	61.6%
	LR-HPV+ **	63	30.0%	62	32.3%		0.0%	125	29.1%
	HPV negative	37	17.6%	2	1.0%	1	3.6%	40	9.3%
	Total	210	100.0%	192	100.0%	28	100.0%	430	100.0%
Women	HR-HPV+ *	23	39.0%	16	57.1%	6	100.0%	45	48.4%
	LR-HPV+ **	24	40.7%	11	39.3%		0.0%	35	37.6%
	HPV negative	12	20.3%	1	3.6%		0.0%	13	14.0%
	Total	59	100.0%	28	100.0%	6	100.0%	93	100.0%
Men	HR-HPV+ *	87	57.6%	112	68.3%	21	95.5%	220	65.3%
	LR-HPV+ **	39	25.8%	51	31.1%		0.0%	90	26.7%
	HPV negative	25	16.6%	1	0.6%	1	4.5%	27	8.0%
	Total	151	100.0%	164	100.0%	22	100.0%	337	100.0%

**Table 6:** Risk of developing AIN1+ or AIN2+ lesions according to the positivity of specific HR HPV genotypes (HPV 16-45-58; or at least one of them).

	AIN1+ *n* (%)	AIN1- *n* (%)	aOR ^	95% CI	AIN2+ *n* (%)	AIN2- *n* (%)	aOR ^	95% CI
**HPV 16 negative**	157 (47.2)	176 (52.8)	Ref.		13 (3.9)	320 (96.1)	Ref.	
**HPV 16 positive**	63 (64.9)	34 (35.1)	2.12 *	1.30–3.46	15 (15.5)	82 (84.5)	4.85 *	2.14–11.02
**HPV 45 negative**	200 (50.2)	198 (49.8)	Ref.		23 (5.8)	375 (94.2)	Ref.	
**HPV 45 positive**	20 (62.5)	12 (37.5)	1.57	0.74–3.36	5 (15.6)	27 (84.4)	3.11 *	1.05–9.19
**HPV 58 negative**	194 (50.9)	187 (49.1)	Ref.		19 (5.0)	362 (95.0)	Ref.	
**HPV 58 positive**	26 (53.1)	23 (46.9)	1.05	0.57–1.96	9 (18.4)	40 (81.6)	4.33 *	1.70–11.01
**Neither of 16-45-58 HR-HPV genotypes**	130 (46.6)	149 (53.4)	Ref.		6 (2.1)	273 (97.9)	Ref.	
**At least one of** **them**	90 (59.6)	61 (40.4)	1.76 *	1.15–2.70	22 (14.6)	129 (85.4)	8.58 *	3.24–22.70

^ Logistic regression model adjusted for sex, age, HIV status, sexual orientation, and HPV vaccination. * Statistically significant (*p* < 0.05).

**Table 7:** Risk of developing AIN1+ or AIN2+ lesions in people with at least one of specific HR HPV genotypes (HPV 16-45-58) and different number of total HR HPV genotypes.

HR-HPV number	AIN1+ *n* (%)	AIN1- *n* (%)	aOR ^	95% CI	AIN2+ *n* (%)	AIN2- *n* (%)	aOR ^	95% CI
**0**	130 (46.6)	149 (53.4)	Ref.		6 (2.1)	273 (97.9)	Ref.	
**1–2**	44 (51.2)	41 (48.2)	1.25	0.76–2.07	13 (15.3)	72 (84.7)	8.53 *	3.04–24.00
**>=3**	46 (69.7)	20 (30.3)	3.00 *	1.61–5.58	9 (13.6)	57 (86.4)	8.65 *	2.70–27.77

^ Logistic regression model adjusted for sex, age, HIV status, sexual orientation, and HPV vaccination. * Statisticallysignificant (*p* < 0.05).

**Table 8:** Risk of developing AIN1+ lesions according to the number of positive low-risk (LR) and high-risk (HR) HPV genotypes. Logistic regression model adjusted for sex, age, HIV status, sexual orientation, and HPV vaccination.

	HR-HPV (*n*)	0	1–2	>=3
LR-HPV (*n*)				
0		Ref.	9.31 * (95% CI: 3.22–27.07)	16.26 * (95% CI: 4.54–58.30)
1–2		8.47 * (95% CI: 2.93–24.40)		
>=3		9.82 * (95% CI: 2.49–38.69)	24.55 * (95% CI: 7.62–79.11)	41.15 * (95% CI: 11.84–142.93)

* Statistically significant (*p* < 0.05).

**Table 9:** Risk of developing AIN2+ lesions according to the number of positive low-risk (LR) and high-risk (HR) HPV genotypes expressed as adjusted Odds Ratios (aORs) with 95% confidence intervals. Logistic regression models adjusted for sex, age, HIV status, sexual orientation, and HPV vaccination.

	AIN2+ *n* (%)	AIN2- *n* (%)	aOR ^	95% CI
**HR-HPV number**				
0	1 (0.6)	164 (99.4)	Ref.	
1–2	17 (9.1)	170 (90.9)	19.96 *	2.55–156.43
>=3	10 (12.8)	68 (87.2)	34.72 *	4.07–296.10
**LR-HPV number in HR-HPV+ patients**				
0	3 (7.9)	35 (92.1)	Ref.	
1–2	9 (3.9)	220 (96.1)	0.53	0.13–2.10
>=3	15 (12.2)	108 (87.8)	1.73	0.45–6.58

^ Logistic regression model adjusted for sex, age, HIV status, sexual orientation, and HPV vaccination. * Statisticallysignificant (*p* < 0.05).

## Data Availability

The data presented in this study are available on request from the corresponding author.
